# PReS-FINAL-2212: Mevalonate kinase deficiency: different faces with separate treatments

**DOI:** 10.1186/1546-0096-11-S2-P202

**Published:** 2013-12-05

**Authors:** P Gençpınar, E Ünsal, B Makay

**Affiliations:** 1 Dokuz Eylül University Hospital, Izmir, Turkey

## Introduction

Mevalonate kinase deficiency-associated periodic fever syndrome is a systemic autoinflammatory disease caused by mutations in the mevalonate kinase gene (MVK), previously named "hyper-IgD syndrome" due to its characteristic increase in serum IgD level. The patients suffer recurrent fever attacks every 2-8 weeks beginning from infancy, often precipitated by immunizations, infections or emotional stress. Fever lasts 2-7 days and can be accompanied by malaise, headache, diarrhea, abdominal pain, vomiting, skin rashes, arthralgia, arthritis, tender lymphadenopathy and hepatosplenomegaly. Fever attacks usually respond to the administration of steroids. However, increasing frequency of fever episodes with steroid use and the natural chronic disease course may require a continuous long-term treatment. Colchicine, cyclosporine, thalidomide and statins are not effective. A TNF-α blocking agent etanercept and IL-1 blocking agents anakinra and canakinumab have been demonstrated to reduce the frequency of fever attacks in MKD. The course and severity of the disease may be quite different.

## Objectives

We report three cases with HIDS, who have separate clinical findings and treatment strategies.

## Methods

Patient 1 was a 12-years-old girl suffered from recurrent fever, abdominal pain, arthritis of the fingers, and cervical lymphadenopathy. The attacks started when she was fifteen months old. Each attack lasted for about a week, 2-3 times annually. She did not have a MEFV mutation. Genetic analysis of the MVK gene revealed two missense mutations: p.V377 on exon 11 and c.38_39 ins TCTG frameshift on exon 2. On follow-up, she had 2 mild attacks in the last year; one of them was treated with a single high dose oral prednisone.

Patient 2 was a 17-months-old boy suffered from periodic fever since two months following birth once or twice a month, lasting for 2-4 days. He had diarrhea and cervical lymphadenopathy during attacks. The heterozygous V726A variant of MEFV was detected. He was put on colchicine therapy; however he did not respond despite 6 months of therapy. Further genetic analysis revealed two mutations on MVK gene (Exon 8. c.803T>C (p.268I>T), and Exon 10 c.1129 G>A (p.377V>I). Simvastatin, etanercept and anakinra were not effective. Corticosteroid shortened the duration of fever during attacks, however; failed to lower the frequency of attacks. He had a dramatic response to canakinumab.

Patient 3 was the sister of patient 3. Parents were non-consanguineous. She suffered from recurrent fever, diarrhea and cervical lymphadenopathy every month since 3 months of age. She had the same mutations of her brother. Besides, the heterozygous E148Q variant of MEFV was detected. She is given corticosteroid during attacks regarding the parents refuse to use IL-1 blocker because of her small age (2 years old).

## Results

We speculate that the more severe and drug-resistant clinic of these siblings may be due to having additional heterozygous MEFV mutations.

## Disclosure of interest

None declared.

